# Revisiting the concept of a cytopathic viral infection

**DOI:** 10.1371/journal.ppat.1006409

**Published:** 2017-07-20

**Authors:** Nicholas S. Heaton

**Affiliations:** Department of Molecular Genetics and Microbiology, Duke University School of Medicine, Durham, North Carolina, United States of America; Mount Sinai School of Medicine, UNITED STATES

## Introduction

The outcome of a viral infection with respect to cellular fate is a fundamental aspect of viral biology. Different virus families have vastly different interactions with their host cells, but they have long been broadly grouped as causing either persistent or cytopathic infections. Persistent infections are established through a variety of mechanisms, but all result in a long-term infection of the host cell. Other viruses cause a lytic (or cytopathic) infection that leads to cellular death shortly after viral infection. But what if cellular death was not the only outcome of a cytopathic viral infection? What if additional cell fates not only occurred but also were important for viral pathogenesis? The focus of this review will be on the evidence for cellular survival after cytopathic virus infections and the importance of these uncharacterized cell populations.

## Can a cell survive a productive, cytopathic viral infection?

Cells that are infected by cytolytic viruses are thought to die shortly after infection. This cell death can occur as a byproduct of viral replication or by the actions of the immune system. But how would one detect any cells that were able to spontaneously eliminate the virus and “survive” infection? Due to the fact that we detect viral infection by looking for viral protein or nucleic acid, a cell that has “survived” and cleared the virus would essentially be identical to an uninfected cell. Thus, traditional methods of assaying the presence of virus (western blot, reverse transcription PCR [RT-PCR], or immunofluorescence) are not appropriate to ask these types of questions. To look for additional cell fates, a system must be used in which any cell that has experienced viral infection is permanently labeled.

To follow cell fate after infection with the cytopathic influenza A virus (IAV), we and others have generated IAV strains that express the enzyme Cre-recombinase [1, 2]. In conjugation with Cre-responsive transgenic animals that activate a fluorescent reporter only after Cre activity, one can look for the presence of fluorescent cells after the clearance of virus. Independent reports have shown that despite IAV being characterized as a highly cytolytic virus, there are populations of lung epithelial cells that survive both direct viral infection and the host immune response [1, 2]. In addition to the work on IAV, data from the laboratory of Diane Griffin have identified the mechanisms of noncytolytic virus clearance of the normally cytolytic Sindbis virus from neurons [3], although some viral RNA persists (reviewed in [4, 5]). Cells have also been reported to survive parvovirus B19 infection in an autophagy-dependent manner [6]. Thus, it appears that infection with highly diverse cytopathic viruses does not always lead to cellular death but can also generate populations of “survivor” cells.

## What are the biological functions of cells that have survived and cleared viral infection?

If little is known about the presence of cells derived from alternative cell fates, even less is known about their potential functions. It has been proposed that nonlytic clearance of viral infection could be critical for preserving organ or tissue function where there is slow or limited regeneration potential, such as in the central nervous system or in the liver [4, 7]. To identify roles for the cells that survive IAV infection, our group has characterized the mRNA profiles of survivor cells. We observed that survivor cells displayed a unique and stable transcriptional profile, suggesting that the cells acquired an altered phenotype after surviving infection [2]. By specifically depleting survivor cells, we were able to show that survivor cells were increasing inflammation in the lung, which delayed lung epithelium regeneration immediately after viral clearance [2].

We were curious, however, as to why the body would harbor apparently detrimental populations of inflammatory survivor cells. In a subsequent study, we determined that the transcriptionally altered survivor cells caused a profound difference in how the host responded to secondary viral infections [8]. In fact, these cells appear to mediate the temporary window of antigen-independent immunity against unrelated viruses after IAV infection has resolved, which has been described in human populations [9]. Our current model is that early after stopping active viral replication, these cells still have within them some amount of virus-derived products and correspondingly activated pathogen-associated molecular pattern (PAMP) receptors. However, they are no longer subject to antagonism of antiviral signaling pathways as occurs during active infection and are, thus, highly inflammatory. Surviving IAV infection also causes long-term transcriptional changes, which not only give these cells a unique phenotype but causes them to up-regulate different cytokines and chemokines during secondary viral infection. During secondary infection, IAV survivor cells also up-regulate lung surfactants and mucins [8]; these proteins can also have direct antiviral functions [10–12]. Interestingly, survivor cell up-regulation of mucins that can help prevent viral secondary infection may also contribute to increased available carbon sources in the lung, which are known to increase susceptibility to secondary bacterial infection [13].

There are numerous other potential roles for cells derived from unappreciated fates. Since these cells are not being cleared from the body, if they are able to retain and harbor even low amounts of virally derived protein long term, they could be significant contributors to a process like B cell affinity maturation. It is also tempting to speculate that diseases that are associated with viral infection but persist after viral clearance, such as chikungunya-mediated arthritis, could be at least partially due to a population of cells that survived infection and influenced the host in a negative manner. This is certainly not an exhaustive list of potential roles for these cells, and these possibilities aren’t necessarily mutually exclusive.

## Is the idea of a “typical” cell fate an artificial concept derived from work in immortalized cell culture systems?

Because of their rapid growth rates (and indefinite proliferation), immortalized cell culture lines (HeLa, Vero, A549, etc.) have long been the standard medium with which to not only propagate virus but also study the nature of viral infection. As a consequence, many of our conclusions regarding the outcome of infection are based on these experimental systems. But while these cell lines are convenient and easy to work with, it is also well appreciated that immortalized cells are frequently altered relative to corresponding primary tissues [14–17].

Are immortalized cell lines (with questionable relationships to primary cells) appropriate for the study of cellular fates after viral infection? We believe that drawing conclusions about cell fate purely from immortalized cell experiments almost certainly leads to an oversimplification of the potential outcomes of viral infection in vivo. In many cases, the cell fate observed in cell culture likely represents the predominant cellular fate in vivo, such as persistent infection after hepatitis C virus (HCV) infection. With some viruses, however, the in vitro cellular fate in cell lines may only represent a reasonably small fraction of the fates after viral infection in vivo. Orthomyxo- and paramyxoviral infections are usually cytolytic in immortalized cells [18–21]. However, after infection of more physiologically relevant air–liquid interface differentiated primary cell cultures, the amount of cell death can be very low [22–25]. Thus, it is important to not overinterpret experiments done in cell culture as necessarily representative of in vivo infection.

## Conclusions and future questions

**Fig 1:**
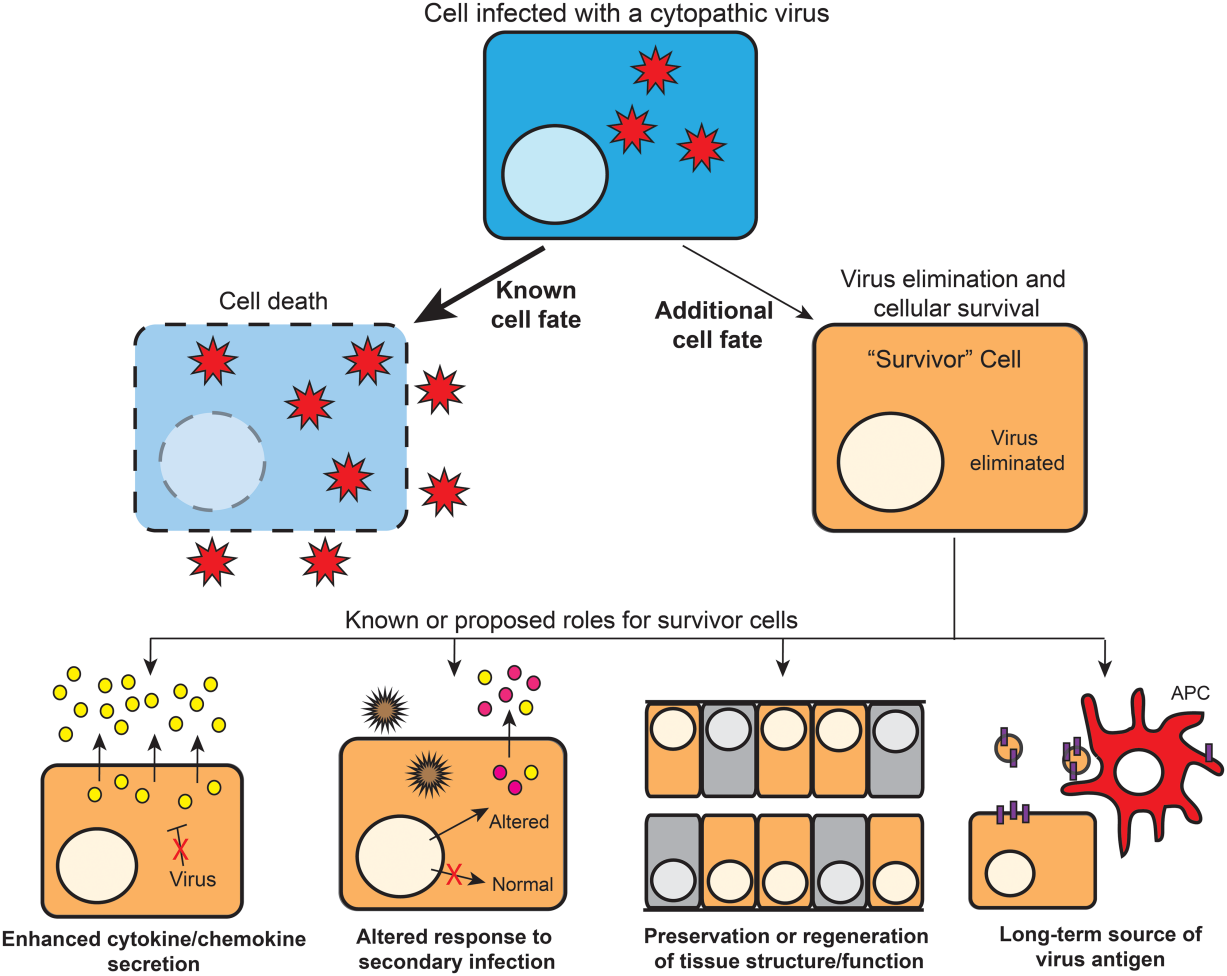
Additional cell fates after viral infection and roles for survivor cells. Cells that are infected with a cytopathic virus that normally causes cell lysis (blue) can sometimes intrinsically eliminate the virus and become a “survivor” cell (orange). These survivor cells have been shown to be important for contributing to inflammation and altering the response to secondary infection. Previously proposed or possible additional functions of survivor cells include (among others): serving as a long-term source of viral protein to antigen-presenting cells (APCs) and/or maintaining critical tissue structures and functions.

There is clearly emerging evidence that some cells can eliminate cytotoxic viral infections and survive (Fig 1). However, these reports are rare when compared to descriptions of the well-studied infection outcome of cell death. The question therefore becomes: Are these additional cell fates rare events and restricted to a few viruses, or are they a more general phenomenon during viral infection that simply has not been characterized? We strongly suspect that for almost every (if not every) virus, additional and uncharacterized cell fates occur and that these cell populations are important for viral replication and disease. Little is known about these potential cell fates after viral infection, in no small part because it is technically difficult to identify such populations. Clearly, the development of experimental systems to look for these cell populations is a necessary first step toward exploring this field.

For the populations of cells derived from alternative cell fates that have already been identified (as well as those that will be identified in the future), there are numerous open questions. The first and most obvious question is: What are the relative frequencies of the different cell fates for a given virus? It is also important to understand the nature of the viral infection that leads to these additional cell fates. Differences in the intrinsic properties of the infecting virus (such as mutations or incompletely packaged viral genomes) could be drivers of additional cell fates. While we typically focus on viral infection generating new infectious virions, many viruses (especially viruses with segmented genomes) produce large numbers of defective or semi-infectious particles in addition to the fully infectious progeny virions (reviewed in [26, 27]). It is reasonable, therefore, to ask: Are “survivor” cell populations partially or completely derived from a nonproductive infection with a defective viral particle? While the experiments are yet to be done, we suspect that a combination of both productive and nonproductive infections can lead to the formation of survivor cell populations under different conditions. We know that semi-infectious or defective viral particles can have significant effects on viral fitness, transmissibility, and the immune response [28–31]. It is certainly a possibility that some of the observed effects could be the result of defective particle “infections” of cells and the establishment of survivor cell populations.

In addition to viral drivers of alternative viral fates, the host response to viral infection almost certainly plays a role in survivor cell formation. No specific host pathway capable of mediating complete viral clearance after the virus has established replication has been reported, and the pathways required for viral clearance are likely different for every virus. There may be as-of-yet undescribed pathways capable of cell-intrinsic viral clearance or alternative versions of known pathways such as autophagy that can mediate clearance. There may also be heterogeneity in the magnitude or the kinetics of the induction of the interferon response across a population of cells that underlies fate decisions. It is also important to note that cellular survival in vivo is more complicated than simply surviving the direct effects of the virus. These antigen-positive cells also must survive clearance by the adaptive immune system to become long-term survivor cells.

In conclusion, there is much work to be done to lay the groundwork for even a basic understanding of the cellular populations that survive cytopathic viral infection. But after this foundation has been laid, we will be able to perform the truly exciting experiments to understand exactly how they are formed and how they contribute to viral pathogenesis. These studies will illuminate an almost completely unexplored aspect of viral infection and will allow novel insights into viral–host interactions.
